# Loss of CD28 on Peripheral T Cells Decreases the Risk for Early Acute Rejection after Kidney Transplantation

**DOI:** 10.1371/journal.pone.0150826

**Published:** 2016-03-07

**Authors:** Burç Dedeoglu, Ruud W. J. Meijers, Mariska Klepper, Dennis A. Hesselink, Carla C. Baan, Nicolle H. R. Litjens, Michiel G. H. Betjes

**Affiliations:** Department of Internal Medicine, section Nephrology and Transplantation, Erasmus Medical Center, Rotterdam, South Holland, the Netherlands; University of California Los Angeles, UNITED STATES

## Abstract

**Background:**

End-stage renal disease patients have a dysfunctional, prematurely aged peripheral T-cell system. Here we hypothesized that the degree of premature T-cell ageing before kidney transplantation predicts the risk for early acute allograft rejection (EAR).

**Methods:**

222 living donor kidney transplant recipients were prospectively analyzed. EAR was defined as biopsy proven acute allograft rejection within 3 months after kidney transplantation. The differentiation status of circulating T cells, the relative telomere length and the number of CD31^+^ naive T cells were determined as T-cell ageing parameters.

**Results:**

Of the 222 patients analyzed, 30 (14%) developed an EAR. The donor age and the historical panel reactive antibody score were significantly higher (p = 0.024 and p = 0.039 respectively) and the number of related donor kidney transplantation was significantly lower (p = 0.018) in the EAR group. EAR-patients showed lower CD4^+^CD28null T-cell numbers (p<0.01) and the same trend was observed for CD8^+^CD28null T-cell numbers (p = 0.08). No differences regarding the other ageing parameters were found. A multivariate Cox regression analysis showed that higher CD4^+^CD28null T-cell numbers was associated with a lower risk for EAR (HR: 0.65, p = 0.028). In vitro, a significant lower percentage of alloreactive T cells was observed within CD28null T cells (p<0.001).

**Conclusion:**

Immunological ageing-related expansion of highly differentiated CD28null T cells is associated with a lower risk for EAR.

## Introduction

Loss of renal function leads to retention of uremic molecules and cytokines, which creates oxidative stress and inflammation. [1] The resulting pro-inflammatory uremic environment underlies the dysfunctional T-cell immunity of end-stage renal disease (ESRD) patients. [2] The major changes in the peripheral T-cell composition are T-lymphopenia, increased T-cell differentiation and loss of telomere length, the latter indicating a history of enhanced T-cell replication. [3]

The T-lymphopenia is largely due to a loss of naive (antigen-inexperienced) T cells, which show signs of increased activation and are more prone to apoptosis. [3] This loss of circulating naive T cells runs in parallel with a decrease in newly formed naive T cells, known as recent thymic emigrants (RTEs, indicating a premature involution of the thymus). In combination with an expanded, more differentiated memory T-cell compartment, this leads to a relatively large decrease in the percentage of circulating naive T cells. [3, 4] The highly differentiated memory T cells are characterized by a loss of the co-stimulatory molecule CD28, making them less dependent on co-stimulation to become activated. [5] Moreover, these cells are known to have a reduced telomere length due to their numerous cell divisions. [3, 6, 7]

The uremia-associated changes in the composition of the peripheral T-cell compartment resemble the physiological changes in the ageing immune system of elderly healthy individuals, [8–10] which leads to the concept of ESRD-related premature immunological ageing. This was confirmed when a combined analysis of the thymic output, differentiation status and the telomere length of T cells in ESRD patients was performed and the results were compared to healthy individuals over a wide age range. [3] A consistent pattern of premature immunological ageing was observed with a discrepancy of 15–20 years between the immunological age of T cells of ESRD patients compared to their chronological age. [3, 11] This prematurely aged T-cell system of ESRD patients offers at least a partial explanation for the increased susceptibility to infections [12], reduced vaccination response [13–16], increased prevalence of malignancies [17, 18] and may also be a non-classical risk factor for cardiovascular diseases. [19–22]

A prematurely aged T-cell system leading to impaired T-cell immunity may also reduce the risk for acute rejection after kidney transplantation, but this has not been systematically studied. In addition, most studies that have assessed the circulating T-cell compartment in relation to acute rejection have only demonstrated percentages of cells. [23, 24] This can lead to erroneous conclusions given the complex changes in all T-cell subsets and for example expansion of memory T cells may be interpreted as a reduction in the number of naive T cells and vice versa.

In this study, we hypothesized that the degree of premature T-cell ageing, based on the absolute number of differentiated T cells, thymic output and telomere length, prior to kidney transplantation (KT) is associated with the risk for early acute allograft rejection (EAR) in kidney transplant recipients. Based upon our analyses we observed that T-cell differentiation status was associated with the risk for EAR after KT.

## Materials and Methods

### Study population

All patients participated in a randomized-controlled clinical trial with the primary aim to study the efficacy of a genotype-based approach to tacrolimus dosing (Dutch trial registry number NTR 2226; http://www.trialregister.nl/trialreg/index.asp). All patients gave written informed consent to participate in the clinical trial, as well as for the sub-study, which is presented here. None of the transplant donors were from a vulnerable population and all donors or next of kin provided written informed consent that was freely given. The study was approved by the Medical Ethical Committee of the Erasmus MC (MEC number 2010–080, EudraCT 2010-018917-30). This study was conducted in accordance with the Declaration of Helsinki.

All patients undergoing a living-donor KT in the period from 1 November 2010 to 1 October 2013 were considered for participation in this study. This study included all ESRD patients with various causes of chronic kidney disease (CKD) (Table 1). Patients were excluded if they were younger than 18 years and if they received immunosuppressive medication (except for glucocorticoids) within 28 days prior to transplantation.

**Table 1:** Patient characteristics. Data are presented as medians (interquartile range).

KT Patients (n = 222)	No Rejection (n = 192) (86%)	Early Rejection (n = 30) (14%)	P
Age recipient	57 (46–64)	55 (47–63)	0.60
Age Donor	52 (40–62)	58 (50–65)	**0.024**
Male gender recipient	118 (61%)	14 (63%)	0.84
Male gender donor	94 (49%)	13 (43%)	0.57
CMV-seropositivity recipient	118 (61%)	15 (50%)	0.23
CMV-serostatus donor/recipient			
-/-	42 (22%)	10 (33%)	0.17
-/+	38 (20%)	5 (17%)	0.69
+/-	32 (17%)	5 (17%)	1.00
+/+	80 (42%)	10 (33%)	0.39
Anti-CMV IgG titer recipient (AU/mL)	65 (42–105)	58 (47–86)	0.75
Mismatch HLA class I	2 (2–3)	3 (2–3)	0.15
Mismatch HLA class II	1 (1–2)	1 (1–2)	0.31
Mismatch HLA class I and II	4 (3–5)	4 (3–5)	0.11
PRA current (%)	0 (0–4)	0 (0–4)	0.52
PRA historic (%)	4 (0–4)	4 (0–29)	**0.039**
Amount of KT	1 (1–1)	1 (1–1)	0.63
Warm ischemia time	20 (16–24)	21 (16–25)	0.69
Cause of CKD			
Nephrosclerosis/atherosclerosis/hypertension	44 (23%)	7 (23%)	0.96
Primary glomerulopathies	26 (14%)	4 (13%)	1.00
Diabetes	41 (21%)	2 (7%)	0.06
Urinary tract infections/stones	5 (3%)	1 (3%)	0.59
Reflux nephropathy	9 (5%)	1 (0%)	1.00
Polycystic Kidney Disease	33 (17%)	7 (23%)	0.42
Other	26 (14%)	5 (17%)	0.58
Unknown	8 (4%)	3 (10%)	0.17
Pre-emptive KT	75 (39%)	17 (57%)	0.07
Genetically-related KT	82 (43%)	6 (20%)	**0.018**
Acute rejection type			
Cellular rejection		25 (83%)	
Antibody-mediated rejection		1 (3%)	
Mixed rejection		4 (13%)	

All patients received induction therapy with basiliximab (20 mg i.v. on day 0 and day 4), tacrolimus (aiming for predose concentrations of 10–15 ng/mL in weeks 1–2, 8–12 ng/mL in weeks 3–4, and 5–10 ng/mL, thereafter), mycophenolate mofetil (starting dose of 1 g b.i.d., aiming for predose concentrations of 1.5–3.0 mg/L), and glucocorticoids. All patients received 50 mg prednisolone b.i.d. intravenously on days 0–3. Thereafter, 20 mg oral prednisolone was started and subsequently tapered to 5 mg at month 3.

We determined clinical variables such as age at time of transplantation, gender, CMV-seropositivity, anti-CMV IgG titer, human leukocyte antigen (HLA) class I and class II mismatches, current and historical panel reactive antibody (PRA) score, warm ischemia time (WIT), number of previous kidney transplantations, preemptive KT (defined as receiving a kidney before the start of renal replacement therapy (RRT)) and related KT (defined as receiving a kidney from a genetically related donor) (Table 1). The HLA-typing was assessed according to the international standards (American Society for Histocompatibility and Immunogenetics/the European Federation for Immunogenetics) using serologic and DNA-based techniques. The PRAs were determined at the laboratory of the blood bank in Leiden, the Netherlands. In all transplantations the complement dependent cross match was negative, but flowcytometry based cross matches were not performed.

We defined EAR as the development of biopsy-proven acute allograft rejection according to the Banff criteria [25] within 3 months after KT.

### PBMCs isolation

By using Ficoll-Paque Plus (GE healthcare, Uppsala, Sweden), peripheral blood mononuclear cells (PBMCs) were isolated from heparinized blood samples drawn from KT-recipients the day before KT. The isolated PBMCs were stored at -150°C with a minimum amount of 10×10^6^ cells per vial for further experiments.

### Absolute numbers of CD4^+^ and CD8^+^ T cells and T-cell differentiation status by FACS analysis

To determine the absolute numbers of the different lymphocyte populations from blood, a Trucount staining was done. In this protocol, 20 μl of the 6-color TBNK reagent (BD Multitest^™^, BD, Erembodegem, Belgium) was used in combination with a BD Trucount^™^ tube (BD) and 50 μl of EDTA blood. This tube contains a number of beads (i.e. bead count; lot-specific) and enables calculation of absolute numbers of cells per μl of blood. The 6-color TBNK reagent contains phycoerythrin (PE)-labeled anti-CD45, AmCyan-labeled anti-CD19, PE-Cy7-labeled anti-CD3, Peridinin chlorophyll (PerCP)-labeled anti-CD4, fluorescein isothiocyanate- (FITC) labeled anti-CD8 and allophycocyanin-Cy7- (APC-Cy7) labeled anti-CD16/CD56.

In addition, a whole blood staining was performed to determine the T-cell differentiation status. [3, 26] Briefly, whole blood was stained with AmCyan-labeled anti-CD3 (BD) in combination with pacific blue-(PB) labeled anti-CD4 (BD) and APC-Cy7-labeled anti-CD8 (BD). T cells were defined as CD4^+^ or CD8^+^ and further defined into four different subsets based on their expression of CCR7 and CD45RO after staining using FITC-labeled anti-CCR7 (R&D systems, Uithoorn, The Netherlands) and APC-labeled anti-CD45RO (BD). Naive T cells were identified as CCR7^+^ and CD45RO^-^, central memory (CM) cells as CCR7^+^ and CD45RO^+^, effector memory (EM) cells as CCR7^-^ and CD45RO^+^ and the highly differentiated effector memory CD45RA^+^ (EMRA) cells as CCR7^-^ and CD45RO^-^. T-cell differentiation is associated with loss of CD28 expression on the cell surface. Numbers of CD28^-^ (or CD28null) T cells within the T-cell subsets were determined by staining with PerCP-Cy5.5-labeled anti-CD28 (BD). Recent thymic emigrants (RTEs) were identified by the expression of CD31 within the naive T-cell pool upon staining with PE-labeled anti-CD31 (Biolegend, Europe BV, Uithoorn, the Netherlands). Samples were measured at the FACSCanto II (BD) acquiring at least 5x10^4^ lymphocytes and analyzed using FACS Diva software version 6.1.2 (BD). [3, 26]

### Telomere Length Assay

Flow fluorescent *in situ* hybridization (flow-FISH) was performed to determine the relative telomere length (RTL) of CD4^+^ and CD8^+^ T cells. PBMCs were isolated and stained with either CD4-biotin (Beckman-Coulter, BV, Woerden, the Netherlands) or CD8-biotin (Biolegend) followed by staining with streptavidin-Cy5 (Biolegend). The PBMCs were fixed and permeabilized (Invitrogen Life Technologies, Bleiswijk, the Netherlands) and by using the telomere FITC-labeled PNA-kit (DakoCytomation, Heverle, Belgium) the telomere length was determined. The sub cell-line 1301 of CCRF-CEM (human T-cell leukemia, Sigma-Aldrich, Zwijndrecht, the Netherlands, ECACC catalogue number 85112105) known for its long telomeres, served as an internal positive control. After acquisition of the samples on the FACSCanto II (BD) and analysis using FACS Diva software version 6.1.2 (BD), the RTL of the CD4^+^ and CD8^+^ T cells was calculated through the next formula [26, 27]:

$$RTL=\frac{\left(\text{median FL}1\text{ sample cells with probe }-\text{median FL}1\text{ sample cells without probe}\right)\;\times \;\mathrm{DNA index of control}(=\;2)\;\mathrm{cells}}{\left(\text{median FL}1\text{ control cells with probe }-\text{median FL}1\text{ control cells without probe}\right)\;\times \;\mathrm{DNA index of sample}\;(=\;1)\;\mathrm{cells}}\;\times 100$$

### Cytokine producing alloantigen-stimulated T cells

To determine frequencies of alloantigen-specific (cytokine-producing) T cells prior to transplantation, we used the CD137 multi-parameter flowcytometric assay as published recently. [28] PBMCs of a kidney transplant recipient were stimulated in presence of co-stimulation (αCD49d, 1 μg/mL; BD) with or without T cell-depleted (>98% pure) donor PBMCs at a 1(5x10^6^):1(5x10^6^) ratio for 24 hours of which the last 12 hours were in presence of Brefeldin A (Golgiplug, BD) and monensin (Golgistop, BD). A distinction between donor and recipient’s cells is enabled by depleting the donor PBMC for CD3^+^ T cells, i.e. CD3^+^ T cells are derived from the recipient. Subsequently, the cell surface was stained using AmCyan-labeled anti-CD3 (BD), APC-Cy7-labeled anti-CD8 (BD) and PerCP-Cy5.5-labeled anti-CD28 (BD) in order to visualize where the cytokine producing T cells are located. Following fixation and permeabilization, CD137 and cytokines were stained intracellular using APC-labeled anti-CD137 (BD), PE-labeled anti-interferon (IFN)-γ (BD) and FITC-labeled anti-interleukin (IL)-2 (BD). At least 0.5-1x10^6^ viable CD3^+^ T cells were acquired on the FACS Canto II. Alloantigen-specific cytokine producing T cells were corrected for the cytokine signal observed in the absence of donor T-cell-depleted PBMC stimulation. Samples were measured on the FACSCanto II (BD) and analyzed using FACS Diva software version 6.1.2 (BD).

### Statistical analysis

All variables are presented as medians with interquartile ranges. The difference between continuous variables was analyzed with the Mann–Whitney U test. The difference between categorical variables was analyzed either with the Pearson’s chi-squared test or with the Fisher’s exact test depending on the expected values in any of the cells of a contingency table. The latter was used when the expected values were lower than 5 in any of the cells. For the assessment of an association between clinical/immunological variables and the presence of EAR, the Cox proportional hazards model was used. Next to this, a Kaplan-Meier curve was created for the assessment of EAR free survival rate stratified for one of the T-cell ageing parameters. The significance level (p-value) was two-tailed and 0.05 was used for all analyses. Statistical analyses were performed using SPSS^®^ version 21.0 for Windows^®^ (SPSS Inc., IL, USA). T-cell subset graphs were created using GraphPad Prism 5 (CA, USA).

## Results

### Patient characteristics

We enrolled 222 consecutive patients who received a kidney transplant from a living donor. Patient characteristics are shown in Table 1. Of the 222 patients analyzed, 30 (14%) had an EAR. The majority of the rejections were classified as grade II (67%), 30% were classified as grade I and only one rejection was classified as grade III. The median age of the patients was 57 years and the median donor age was 53 years. The donors were significantly older in the EAR group with a median age of 58 years compared to a median age of 52 years in the no rejection group (p = 0.024). The majority of the patients (92%) received a donor kidney for the first time, 14 patients (6%) for the second time and three patients (1%) for the third time. Of the 30 patients who developed EAR, 11 (37%) received T-cell depletion therapy consisting of either alemtuzumab subcutaneously or rabbit anti-thymocyte globulin (rATG) intravenously. Four patients had a transplantectomy within the first 3 months after transplantation. Two of these patients had a therapy-resistant cellular acute rejection within the first week after KT. The other two patients were in the no rejection group and the graft was removed due to vascular problems that occurred during the transplantation procedure.

The historical PRA score was significantly higher in the EAR group compared with the no rejection group (p = 0.039). The relative number of genetically related KT was significantly lower in the EAR group (20% vs 43%, p = 0.018). Other potential risk factors for acute rejection, like number of HLA mismatches or previous kidney transplantation, did not associate with EAR in this patient group.

### Patients with EAR have a lower number of CD4^+^CD28null T cells prior to KT

**Fig 1:**
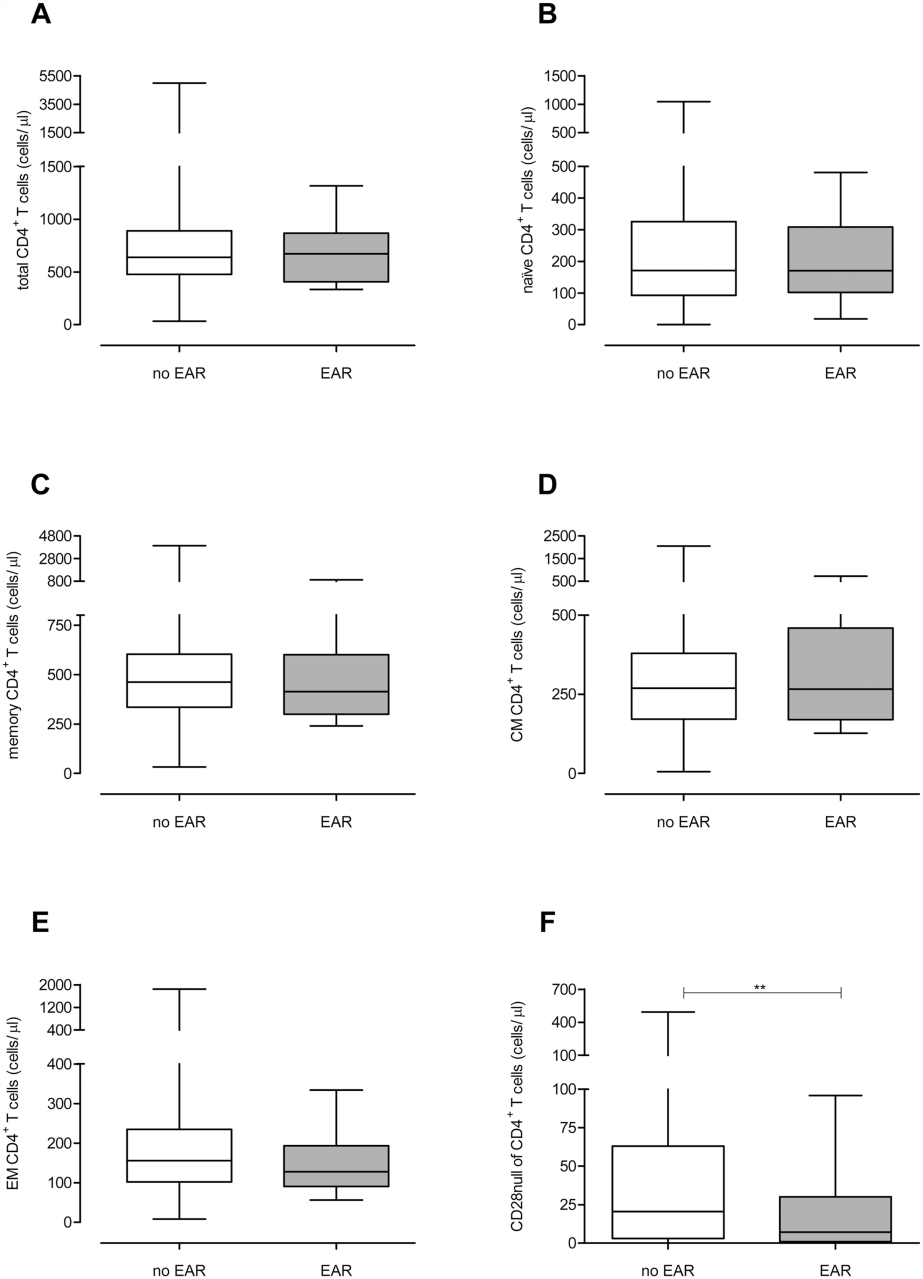
CD4^+^ T-cell differentiation status prior to KT. Absolute numbers of (A) total, (B) naive, (C) memory, (D) CM, (E) EM and (F) CD28null CD4^+^ T cells are shown for the no rejection (white boxplot, n = 192) and EAR (grey boxplot, n = 30) group of patients. Significant differences were calculated and shown (*p<0.05, **p<0.01, **p<0.001).

**Fig 2:**
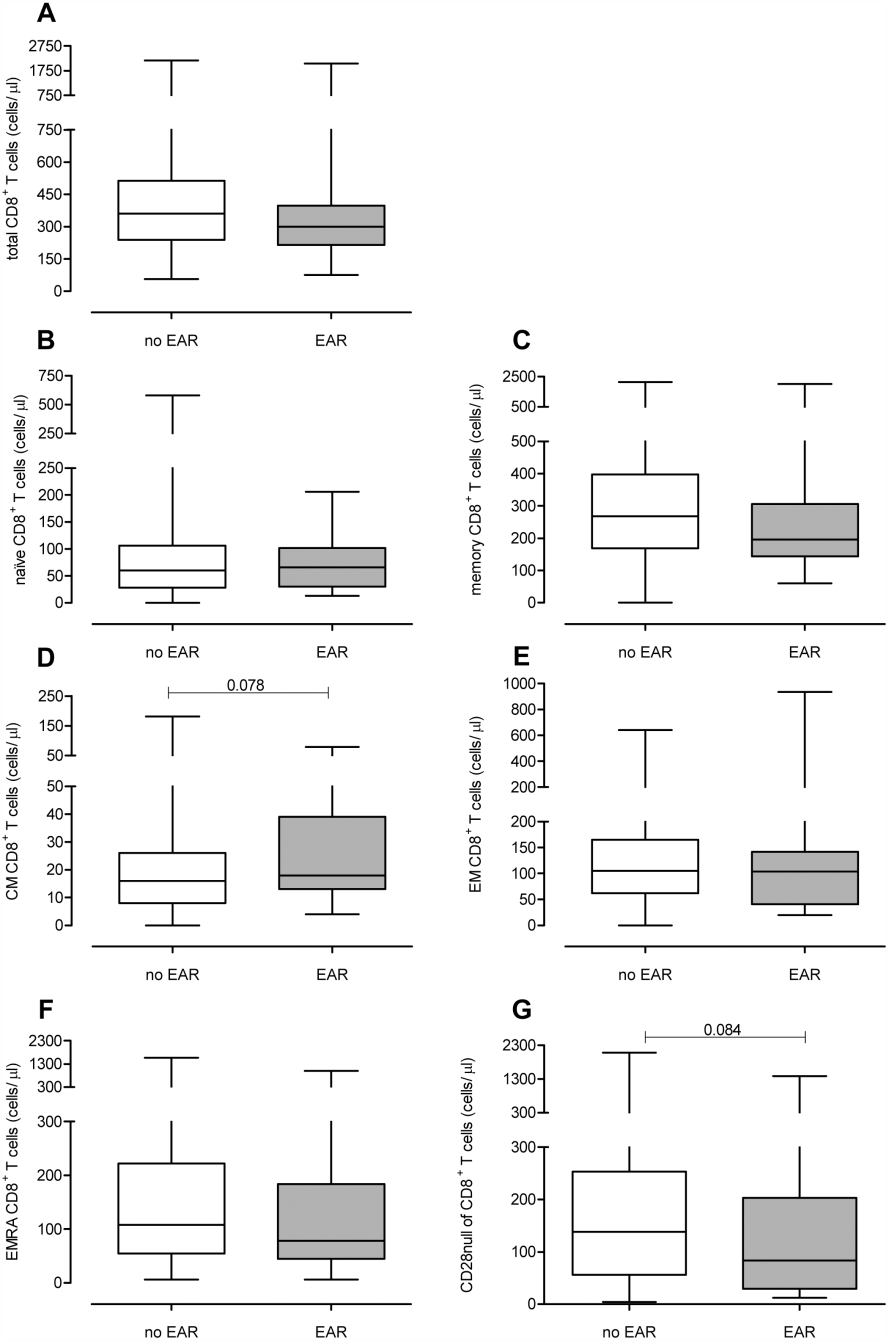
CD8^+^ T-cell differentiation status prior to KT. Absolute numbers of (A) total, (B) naive, (C) memory, (D) CM, (E) EM, (F) EMRA and (G) CD28null CD8^+^ T cells are shown for the no rejection (white boxplot, n = 192) and EAR (grey boxplot, n = 30) group of patients. Significant differences were calculated and shown (*p<0.05, **p<0.01, **p<0.001).

The CD4^+^ and CD8^+^ T-cell differentiation status of both patient groups was determined prior to KT (Fig 1. **CD4**^**+**^ **T-cell differentiation status prior to KT.** Absolute numbers of (A) total, (B) naive, (C) memory, (D) CM, (E) EM and (F) CD28null CD4^+^ T cells are shown for the no rejection (white boxplot, n = 192) and EAR (grey boxplot, n = 30) group of patients. Significant differences were calculated and shown (*p<0.05, **p<0.01, **p<0.001). & Fig 2. **CD8**^**+**^ **T-cell differentiation status prior to KT.** Absolute numbers of (A) total, (B) naive, (C) memory, (D) CM, (E) EM, (F) EMRA and (G) CD28null CD8^+^ T cells are shown for the no rejection (white boxplot, n = 192) and EAR (grey boxplot, n = 30) group of patients. Significant differences were calculated and shown (*p<0.05, **p<0.01, **p<0.001)). In S1 Fig, typical examples of the gating strategy are depicted for the flowcytometric analysis of the CD4^+^ and CD8^+^ T-cell population, respectively. No significant differences were found in the number of CD4^+^ T cells (Fig 1A) between the EAR group and no rejection group. Moreover, the number of naive, memory, CM and EM (Fig 1B–1E) was not significantly different. Interestingly, compared to the no rejection group, the EAR group had significant lower number of CD28null T cells within the CD4^+^ T-compartment (21 cells/μl vs. 7 cells/μl, p<0.01, Fig 1F).

The total number of CD8^+^ was not significantly different between the two patient groups (Fig 2A). The number of naive, memory and CM, EM and EMRA (Fig 2B–2F) CD8^+^ T cells did also not show any significant differences between the two groups. Furthermore, the EAR group tended to have a lower number of CD28null CD8^+^ T cells (p = 0.08, Fig 1G) compared to the no rejection group.

Even though we mainly focused on absolute cell numbers, we also analyzed percentages of the different T cell subsets to provide a better comparison with previous studies. These analyses showed similar findings (S1 Table**: T-cell differentiation status before kidney transplantation in patients with or without rejection within the first 3 months.**). Again, significant higher percentages of CD4^+^CD28null T cells were seen in the no rejection group (p = 0.011). Next to this, (a tendency for) higher percentages of CD4^+^ and CD8^+^ CM T cells were seen in the EAR group (p = 0.055 and p = 0.005 respectively).

### No differences in relative telomere length and RTEs between the EAR group and no rejection group prior to KT

As a marker for the proliferative history, the RTL of the CD4^+^ and CD8^+^ T cells was determined. For both T-cell subsets no significant differences were found between the EAR group and the no rejection group regarding the RTL (Table 2).

**Table 2:** RTL and RTEs content before kidney transplantation in patients with or without rejection within the first 3 months. Data are presented as medians (interquartile range). *RTL*: Relative Telomere Length, *RTEs*: Recent thymic emigrants.

KT Patients (n = 222)	No Rejection (n = 192) (86%)	Early Rejection (n = 30) (14%)	P
RTL of CD4^+^ T cells	12.1 (9.1–15.0)	11.5 (10.0–13.3)	0.90
RTL of CD8^+^ T cells	11.4 (9.2–15.3)	11.1 (10.4–14.4)	0.83
CD31^+^CD4^+^ naive T-cell numbers (/μl)	106.5 (56.7–207.4)	104.3 (77.0–192.9)	0.99
CD31^+^ within CD4^+^ naive T-cells (%)	66.4 (55.1–75.1)	62.2 (53.7–76.8)	0.69
CD31^+^CD8^+^ naive T-cell numbers (/μl)	55.4 (24.1–566.3)	65.0 (12.8–203.9)	0.80
CD31^+^ within CD8^+^ naive T-cells (%)	97.7 (94.4–98.9)	97.9 (93.9–99.5)	0.41

The number and percentages of RTEs were identified by the expression of CD31 within the naive T-cell pool. No significant differences for CD4^+^ or CD8^+^ T cells were found between the two groups prior to KT (Table 2).

### Donor age, historical PRA, a related kidney donation and absolute numbers of CD4^+^CD28null T cells are related with the risk for EAR

The results of the univariate Cox regression analysis of the patient characteristics are presented in Table 3. This analysis showed that receiving an older donor kidney was associated with a higher risk for EAR (HR: 1.41, p = 0.011). Besides this, a higher historical PRA score was also associated with a higher risk for EAR (HR: 1.11, p = 0.001). Receiving a donor kidney from a relative reduced the risk for EAR (HR: 0.36, p = 0.025).

**Table 3:** Hazard ratios for the clinical characteristics in relation to early acute allograft rejection (univariate analysis).

	HR	95%CI	P
Age donor (decades)	1.41	1.09–1.85	**0.011**
PRA historic (%)	1.11	1.04–1.18	**0.001**
Genetically related KT	0.36	0.15–0.88	**0.025**
CD4 positive CD28null T cells (cells/μL)	0.65	0.45–0.95	**0.025**
CD8 positive CD28null T cells (cells/μL)	0.98	0.94–1.03	0.420
CD4 positive central memory T cells (%)	1.02	1.00–1.04	0.124
CD8 positive central memory T cells (%)	1.05	1.00–1.09	**0.035**
CD4 positive CD28null T cells (%)	0.92	0.85–1.00	**0.047**

*P ≤ 0.05, **P ≤ 0.01, ***P ≤ 0.001. CI: confidence interval, HR: hazard ratio. Age of the donor is presented in decades, PRA historic is presented with steps of 5%, and the CD4 positive and the CD8 positive CD28null cells are presented with steps of 20 cells/μL.

The univariate Cox regression analysis of the T-cell subsets showed that a higher number of absolute CD4^+^CD28null T cells, i.e. having a more differentiated CD4^+^ T-cell compartment, was associated with a lower risk for EAR (HR: 0.65, p = 0.025). In contrast, the number of CD8^+^CD28null T cells was not associated with the risk for EAR (HR: 0.98, p = 0.420). Furthermore, higher percentages of CD4^+^CD28null T cells were also associated with a lower risk for rejection (HR: 0.92; p = 0.047). However, higher percentages of CD8^+^ CM T cells, representative of having a less differentiated memory compartment, were associated with a higher risk for EAR (HR: 1.05; p = 0.035).

A multivariate Cox regression analysis was performed with the three aforementioned clinical characteristics (i.e. donor age, historical PRA and a related kidney donation) as covariates (Table 4). In accordance with the univariate analysis, a higher absolute number of CD4^+^CD28null T cells was associated with a lower risk for EAR (HR: 0.65, p = 0.028). Again, no association could be observed between the number CD8^+^CD28null T cells and the risk for EAR (HR: 0.99, p = 0.415). Next to this, only the percentages of CD4^+^CD28null T cells were associated with the risk for EAR (S2 Table**: Hazard ratios for the clinical characteristics in relation to early acute allograft rejection (multivariate analysis).**). Higher percentages of these cells were also associated with a lower risk for EAR (HR: 0.91; p = 0.036).

**Table 4:** HRs for the T-cell parameters in relation to early acute allograft rejection (multivariate analysis).

	HR	95%CI	P
Age donor (decades)	1.40	1.06–1.84	**0.016**
PRA historic (%)	1.13	1.06–1.21	**<0.001**
Genetically related KT	0.47	0.19–1.17	0.102
CD4 positive CD28null T cells (cells/μL)	0.65	0.45–0.96	**0.028**

*P ≤ 0.05,

**P ≤ 0.01,

***P ≤ 0.001. CI: confidence interval, HR: hazard ratio. The CD4 positive and the CD8 positive CD28null cells are presented with steps of 20 cells/μL. **Covariates:** age donor, PRA historic, related KT.

**Fig 3:**
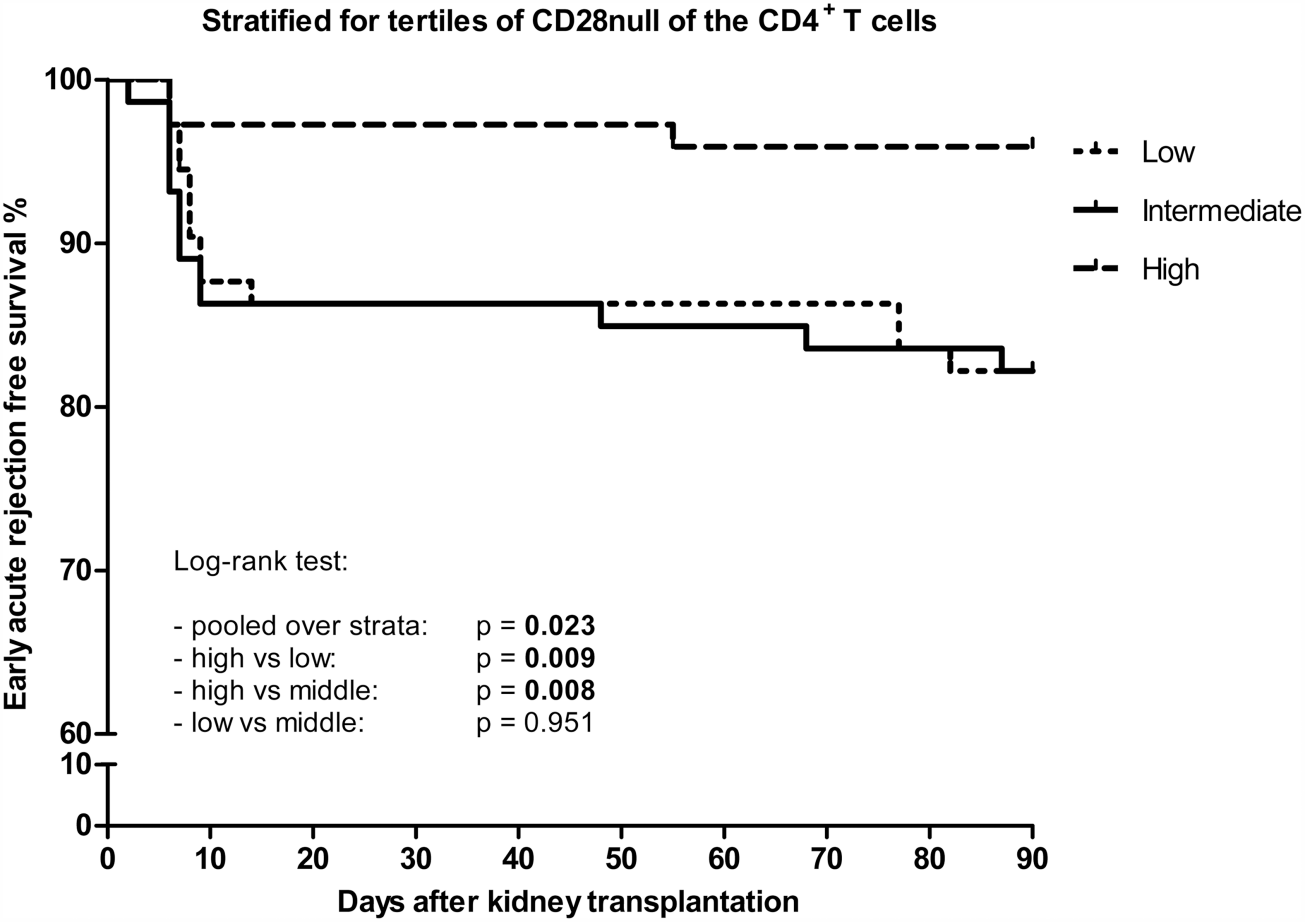
Tertiles of CD4^+^CD28null T cells and EAR free survival. Kaplan-Meier curve representing EAR free survival rate against time after kidney transplantation in days. The curves represent the tertiles of CD4^+^CD28null T cells. Low represents <4.33 cells/μl, intermediate represents 4.33–36.67 cells/μl and high represents >36.67 cells/μl. These groups were created based upon the frequencies of the cell numbers in our cohort and were divided into three equal groups.

Furthermore, we generated a Kaplan-Meier curve with the EAR freee survival stratified for the tertiles of CD4^+^CD28null T cells (Fig 3**: Tertiles of CD4**^**+**^**CD28null T cells and EAR free survival.** Kaplan-Meier curve representing EAR free survival rate against time after kidney transplantation in days. The curves represent the tertiles of CD4^+^CD28null T cells. Low represents <4.33 cells/μl, intermediate represents 4.33–36.67 cells/μl and high represents >36.67 cells/μl. These groups were created based upon the frequencies of the cell numbers in our cohort and were divided into three equal groups). This analysis showed that high numbers of these cells were correlated with a significant higher EAR free survival rate compared with intermediate and low numbers of these cells (p = 0.008 and p = 0.009 respectively).

### Alloantigen-specific T-cells predominantly co-express CD28

**Fig 4:**
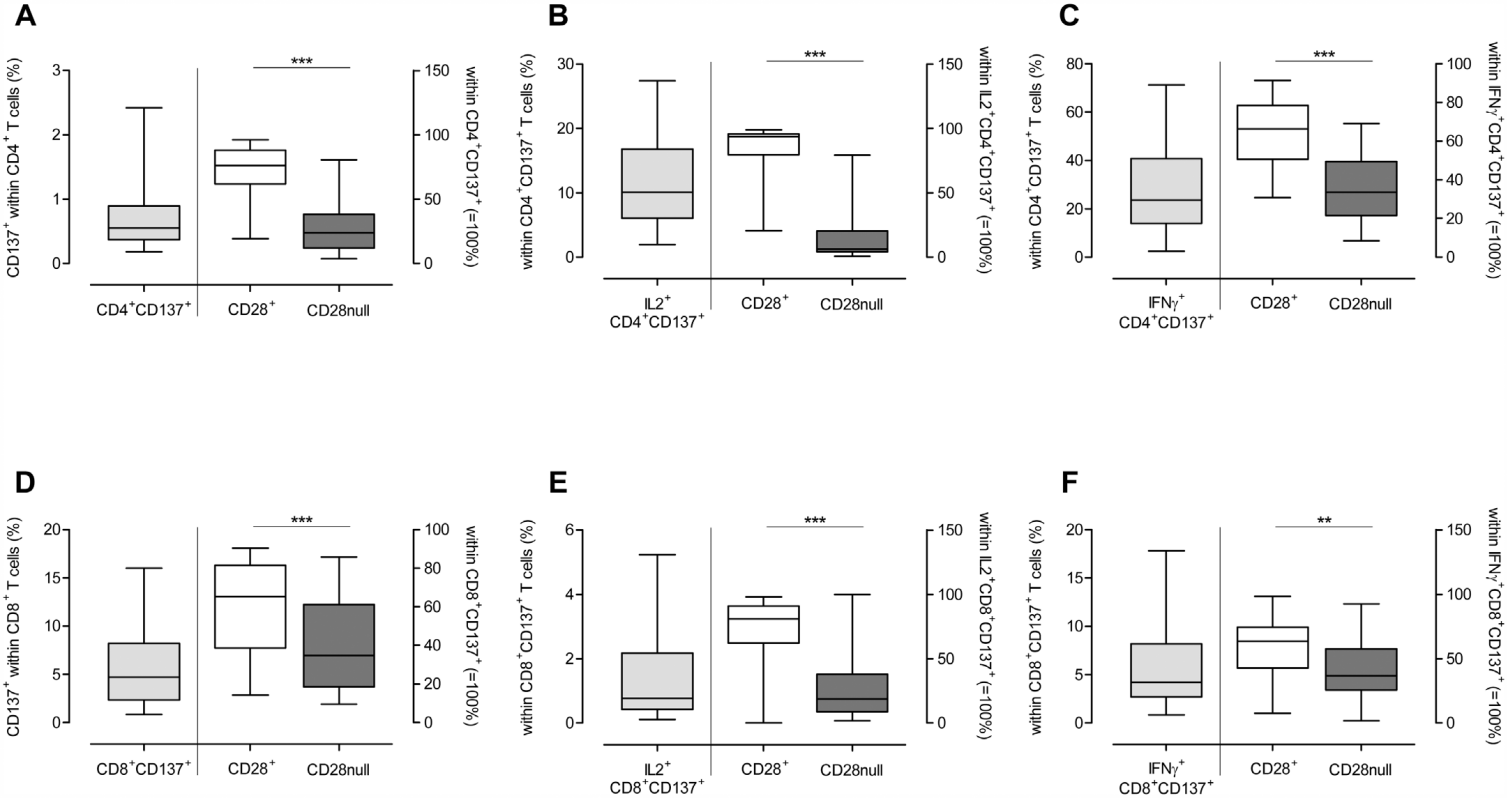
Cytokine producing alloantigen-stimulated T cells. First, the frequency of CD137^+^ cells within CD4^+^ T-cell population was determined (grey boxplot) in ESRD patients. These cells were divided into (A) a CD28^+^ (white boxplot) and a CD28null subset (black boxplot). Next, the frequency of IL-2^+^ cells within the CD137^+^CD4^+^ was determined (grey boxplot). Furthermore, these cells were divided into (B) a CD28^+^ (white boxplot) and a CD28null subset (black boxplot). Also the frequency of IFN-γ^+^ CD137^+^CD4^+^ T cells was determined (grey boxplot) and also these cells were divided into (C) a CD28^+^ (white boxplot) and a CD28null subset (black boxplot). Next to the CD4^+^, the frequency of CD137^+^ cells within the CD8^+^ T-cell population was determined (grey bar) and divided into a CD28^+^ (white boxplot) and a CD28null subset (black boxplot) (D). Within these CD137^+^CD8^+^ T cells, the frequency of IL-2 (F) and IFN-γ^+^ (G) was determined (grey boxplot) and divided into a CD28^+^ (white boxplot) and a CD28null subset (black boxplots) (E+F). Significant differences were calculated and shown (*p<0.05, **p<0.01, **p<0.001).

To obtain more insight between the association of highly differentiated T cells and the risk for EAR, a multiparameter flowcytometric assay (i.e. combining intracellular cytokine staining with cell surface markers) [29] was performed upon alloantigen stimulation of PBMCs obtained from kidney transplant recipients prior to kidney transplantation (Fig 4**: Cytokine producing alloantigen-stimulated T cells.** First, the frequency of CD137^+^ cells within CD4^+^ T-cell population was determined (grey boxplot) in ESRD patients. These cells were divided into (A) a CD28^+^ (white boxplot) and a CD28null subset (black boxplot). Next, the frequency of IL-2^+^ cells within the CD137^+^CD4^+^ was determined (grey boxplot). Furthermore, these cells were divided into (B) a CD28^+^ (white boxplot) and a CD28null subset (black boxplot). Also the frequency of IFN-γ^+^ CD137^+^CD4^+^ T cells was determined (grey boxplot) and also these cells were divided into (C) a CD28^+^ (white boxplot) and a CD28null subset (black boxplot). Next to the CD4^+^, the frequency of CD137^+^ cells within the CD8^+^ T-cell population was determined (grey bar) and divided into a CD28^+^ (white boxplot) and a CD28null subset (black boxplot) (D). Within these CD137^+^CD8^+^ T cells, the frequency of IL-2 (F) and IFN-γ^+^ (G) was determined (grey boxplot) and divided into a CD28^+^ (white boxplot) and a CD28null subset (black boxplots) (E+F). Significant differences were calculated and shown (*p<0.05, **p<0.01, **p<0.001)). In S2 Fig a representative example of the gating strategy is shown of the dissection of alloantigen-specific (CD137^+^ and CD137^+^ IL-2^+^ or IFN-γ^+^) CD4^+^ T cells into a CD28null- or CD28^+^ T-cell compartment. A similar FACS-analysis was performed for the CD8^+^ T-cell population. All CD3^+^ T cells are of patient’s origin, as the donor cells were depleted of T cells prior to stimulation.

First, the frequency of alloantigen-specific CD137-expressing T cells was determined in the CD4^+^ T-cell compartment and these were mainly CD28^+^ (i.e. 72.4% vs 27.6%, p<0.001, Fig 4A). In addition, the CD28^+^ T cells contained more IL-2 (86.9% vs 13.1%, p<0.001) and IFN-γ (i.e. 65.1% vs. 34.9%, p<0.001) producing alloantigen-specific T cells compared to the CD28null fraction (Fig 4B and 4C) upon alloantigen-stimulation.

Similar results were found for the CD8^+^ T cells. Upon alloantigen-stimulation, the CD137^+^CD8^+^ T-cells were mainly located within the CD28^+^ T cells (i.e. 60.4% vs 39.6%, p<0.001, Fig 4D). A higher proportion of these cells were also able to produce more IL-2 compared to their CD28null counterparts (i.e. 72.4% vs 27.6%, p<0.001) and IFN-γ (i.e. 57.4% vs 42.6%, p<0.01) (Fig 4E and 4F).

## Discussion

In this study we analyzed within a large homogenous cohort of patients whether the degree of premature T-cell ageing prior to KT is associated with the risk for EAR post-KT. Of the three T-cell ageing parameters (thymic output, differentiation status and telomere length) used for the assessment of an immunological T-cell age, only the differentiation status was associated with the risk for EAR. A higher number as well as percentage of CD28null T cells, mainly within the CD4^+^ T-cell population, is associated with a lower risk for EAR. The number of RTEs or the relative telomere length of CD4^+^ and CD8^+^ T cells were both not associated with the risk for rejection.

CD28null T cells are predominantly located within the (antigen-experienced) memory population and in particular within the more differentiated T cells. [30] Loss of CD28 on the cell surface of (CD4^+^) T cells is one of the features of T-cell ageing as CD28null T cells are present at a low frequency and rarely found in young individuals. Moreover, CD4^+^CD28null T cells are highly associated with seropositivity for cytomegalovirus (CMV). [31–33] However, in this study we did not observe a significant difference with respect to frequencies of CMV-seropositive individuals between the EAR group and the no rejection group.

The majority of CD28null T cells lack the expression of CCR7. This is important for homing to secondary lymphoid organs [34] where (naive) T cells are activated by antigen-presenting cells presenting alloantigens in a direct or indirect manner. In addition to the lack of CD28, they are less able to provide co-stimulation through the CD40L-CD40 pathway contributing to defective helper function. [35]

A characteristic feature of CD4^+^CD28null T cells is their restricted T-cell receptor profile [36, 37] compared to the CD28^+^ T-cell population. This may compromise their reactivity to foreign antigens like for example alloantigens. Thus, theoretically higher numbers of CD28null T cells might result in lower alloreactivity. This hypothesis is supported by our finding that IL-2 and IFN-γ producing alloantigen-specific T cells were predominately located within the CD28^+^ T-cell population, both within the CD4^+^ and the CD8^+^ T-cell compartment. In addition, in liver transplant recipients, higher frequencies of CD4^+^CD28^+^ T cells were found in allograft rejecting patients. [38] Next to this, the higher percentages of CD4^+^ and CD8^+^ CM T cells in the EAR group, supports this finding by suggesting that the less differentiated cells are more present in patients who develop an acute rejection.

The finding that high CD28null T-cell numbers are associated with a lower risk for allograft rejection is in line with an earlier small study in which these cells were shown to have an exhausted phenotype. [24] Furthermore, several studies support the senescent character of these CD28null T cells. A study by Nunes et al. showed that highly differentiated CD8^+^ T cells were accompanied by an increase of CD28null and CD27^-^ T cells. [39] In line with this, the highly differentiated CD8^+^ T cells were also associated with PD-1 positivity, which was in accordance with replicative senescence of these T cells. [39] Next to this, a study by Dirks et al. showed that CMV viremia was associated with increased PD-1 expression on CD4^+^CD28nullCD27^-^ T cells in transplant recipients. [40] Furthermore, a study by Koch et al. showed that the expression of the senescence markers CD57 and KLRG-1 were more present in the highly differentiated CD4^+^ and CD8^+^ T cells, and were almost absent in the naive T-cell populations. [41] Our group also showed that the percentage of CD57^+^ cells was significantly higher in CD4^+^ memory T cells. [42] In a recent study, a lower frequency of CD4^+^CD28null T cells was also observed in relation to acute rejection within the first year after kidney transplantation but statistical significance was lost in the multivariate analysis. [23] The patient population in that study was much more heterogeneous, including post-mortal kidney transplantations, more re-transplantations and a higher number of CMV-seropositive patients which could at least in part explain the difference in findings between both studies. [23] The higher number of CMV-seropositive patients in that study might also explain why we could not find relations between CD8^+^ EMRA T-cells and the development of EAR in our current study.

Compared to the CD28^+^ T-cell population, the CD28null T cells are known to have shorter telomeres. [31] The fact that we could not detect a correlation between the overall telomere length and the risk for EAR, might be explained by the relatively low frequency of CD28null T cells within the CD4^+^ T-cell population in which the relative telomere length was assessed. In line with our findings, a study by Oetting et al. could also not find an association between the RTL and the risk for acute rejection. [43]

In this study we could not find an association between RTEs and the risk for EAR based on the expression of CD31. Since the thymus involutes rapidly after puberty, the contribution of the thymus to maintain the (naive) T-cell pool is relatively small in older individuals. [44] Maintaining adequate numbers of naive T cells upon ageing mainly relies on homeostatic proliferation either through homeostatic cytokines like IL-7 or low-affinity T-cell receptor interactions with self-antigens being presented by antigen-presenting cells [45]. Since the total number of naive T cells is not different between the two groups of patients, it is likely that the degree of homeostatic proliferation is similar. These findings suggest that the naive T-cell compartment is not of significant importance for alloreactivity within the first three months after KT and that the memory T-cell compartment is more relevant. [46]

Another interesting finding in this study was the higher risk for EAR in patients with a higher historical PRA score. Several other studies have shown that high levels of historical PRA scores are associated with rejection and graft failure. [47–49] Our findings support the results of these studies with regard to rejection and suggest that a peak historical PRA score might also be an important contributor in the setting of EAR in KT.

As the T-cell ageing parameters do not change post-KT [26], it is likely that at the time of rejection the composition of T cells including the frequency of CD28null T cells is similar to the pre-KT value. This means that allograft rejection risk assessment based on T-cell ageing prior to KT probably resembles the T-cell age prior to time of rejection.

In conclusion, the T-cell ageing-related expansion of highly differentiated CD4^+^CD28null T cells in ESRD patients is associated with a lower risk for EAR. This may be related to a significantly lower percentage of alloreactive T cells within the CD28null T cell fraction. This study provides in depth analysis of the various T cell subsets with regard to T-cell ageing in the setting of KT. To give a more accurate representation we mainly focused on absolute cell numbers instead of percentages. Furthermore proliferative history was also taken into account by the assessment of RTL. These characteristics were then combined with functional capacities of the highly differentiated CD28null T cells and associations were drawn with regard to EAR. The patient characteristics of the two patient groups are highly similar, which makes the study population homogenous. Next to this, only living-donor KT was considered. We believe that the combination of the aforementioned aspects contribute novel facets in the relationship between premature T-cell ageing and the risk for EAR after KT. Further analyses are needed to investigate the properties of these cells and to define cut-off values for the clinical practice. In the future this could possibly attribute to an optimal risk assessment for a personalized immunosuppressive regimen.

## Supporting Information

S1 Fig Gating strategy of (A) CD4^+^ and (B) CD8^+^ T cells. A typical example of the gating strategy is shown. Briefly, lymphocytes were identified based on the forward/sideward characteristics followed by the selection of the CD3^+^CD4^+^ T cells. These CD4^+^ T cells were dissected into subsets using CCR7 and CD45RO. Furthermore, the number of CD28null cells was examined within the (A) CD4^+^ T-cell population. The same strategy was followed for (B) CD8^+^ T cells.
(EPS)

S2 Fig Example of the gating strategy of cytokine producing alloantigen-stimulated T cells. Briefly, lymphocytes were identified based on the forward/sideward characteristics. Next the CD3^+^CD4^+^ (i.e. CD8^-^) and CD3^+^CD8^+^ were selected and within these, the CD137^+^ were selected as shown for the CD4^+^ population. These cells were divided into a CD28^+^ and CD28null population. Furthermore the frequency of IL-2^+^ and IFN-γ^+^ CD137^+^CD4^+^ was determined and also dissected into a CD28^+^ and CD28null subset. A similar approach was applied for the CD8^+^ T-cell compartment.
(EPS)

S1 Table T-cell differentiation status before kidney transplantation in patients with or without rejection within the first 3 months. (DOCX)

S2 Table Hazard ratios for the clinical characteristics in relation to early acute allograft rejection (multivariate analysis). (DOCX)
