# Supplementary material for: Loss of CD28 on Peripheral T Cells Decreases the Risk for Early Acute Rejection after Kidney Transplantation
Source: PLoS One. 2016 Mar 7;11(3):e0150826. doi: 10.1371/journal.pone.0150826 (PMC4780739; doi:10.1371/journal.pone.0150826)
Supplement: S1 Table — (DOCX) [file pone.0150826.s003.docx]

|  | **Supplementary Table 1. T-cell differentiation status before kidney transplantation in patients with or without rejection within the first 3 months** | | |  |  |
| --- | --- | --- | --- | --- | --- |
| KT Patients (n = 222) | | No Rejection (n =192) (86%) | Early Rejection (n = 30) (14%) | | P |
| CD4^+^ naive T cells (%) | | 29.4 (18.0 – 42.0) | 29.7 (24.1 – 36.4) | | 0.905 |
| CD4^+^ memory T cells (%) | | 70.6 (60.0 – 82.0) | 70.3 (63.6 – 75.9) | | 0.905 |
| CD4^+^ central memory T cells (%) | | 40.5 (31.3 – 50.6) | 46.6 (37.3 – 54.6) | | 0.055 |
| CD4^+^ effector memory T cells (%) | | 24.4 (16.5 – 32.5) | 24.9 (14.6 – 27.7) | | 0.303 |
| CD4^+^CD28null T cells (%) | | 3.0 (0.5 – 9.6) | 1.0 (0.2 – 4.1) | | **0.011** |
| CD8^+^ naive T cells (%)  CD8^+^ memory T cells (%)  CD8^+^ central memory T cells (%)  CD8^+^ effector memory T cells (%)  CD8^+^ EMRA T cells (%)  CD8^+^CD28null T cells (%) | | 18.1 (8.8 – 35.1)  81.9 (65.0 – 91.2)  4.2 (2.3 – 7.9)  32.7 (20.3 – 45.4)  33.7 (18.5 - 52.1)  40.5 (19.6 - 60.4) | 22.8 (13.8 – 39.9)  77.3 (60.1 – 86.2)  6.1 (4.1 - 13.6)  28.2 (23.8 - 41.6)  31.6 (16.9 - 50.3)  27.6 (19.6 - 52.9) | | 0.323  0.323  **0.005**  0.899  0.759  0.190 |
| Data are presented as medians (interquartile range). | | | | | |
|  | | | | | |
