# Supplementary material for: Loss of CD28 on Peripheral T Cells Decreases the Risk for Early Acute Rejection after Kidney Transplantation
Source: PLoS One. 2016 Mar 7;11(3):e0150826. doi: 10.1371/journal.pone.0150826 (PMC4780739; doi:10.1371/journal.pone.0150826)
Supplement: S2 Table — (DOCX) [file pone.0150826.s004.docx]

| **Supplementary Table 2. Hazard ratios for the clinical characteristics in relation to early acute allograft rejection (multivariate analysis)** | | | |
| --- | --- | --- | --- |
|  | HR | 95% CI | P |
| Age donor (decades)  PRA historic (%)  Genetically related KT  CD4 positive CD28null T cells (%) | 1.43  1.13  0.51  0.91 | 1.09 – 1.88  1.07 – 1.22  0.21 – 1.28  0.84 – 0.99 | **0.011**  **<0.001**  0.152  **0.036** |
| **P* ≤ 0.05, ***P* ≤ 0.01, ****P* ≤ 0.001. CI: confidence interval, HR: hazard ratio. Age of the donor is presented in decades, PRA historic is presented with steps of 5%, the CD4 positive and the CD8 positive CD28null cells are presented with steps of 20 cells/µL, CD4 and CD8 positive central memory T cells are presented with steps of 1%, and the CD4 positive CD28 null T cells are presented with steps of 1% | | | |
